# Towards an Internet of Science

**DOI:** 10.1515/jib-2019-0024

**Published:** 2019-05-30

**Authors:** Jens Allmer

**Affiliations:** Hochschule Ruhr West, University of Applied Sciences, Medical Informatics and Bioinformatics, 45407 Mülheim an der Ruhr, Germany

**Keywords:** workflow management, computational pipelines, internet of things, code smells, scientific computing

## Abstract

Big data and complex analysis workflows (pipelines) are common issues in data driven science such as bioinformatics. Large amounts of computational tools are available for data analysis. Additionally, many workflow management systems to piece together such tools into data analysis pipelines have been developed. For example, more than 50 computational tools for read mapping are available representing a large amount of duplicated effort. Furthermore, it is unclear whether these tools are correct and only a few have a user base large enough to have encountered and reported most of the potential problems. Bringing together many largely untested tools in a computational pipeline must lead to unpredictable results. Yet, this is the current state. While presently data analysis is performed on personal computers/workstations/clusters, the future will see development and analysis shift to the cloud. None of the workflow management systems is ready for this transition. This presents the opportunity to build a new system, which will overcome current duplications of effort, introduce proper testing, allow for development and analysis in public and private clouds, and include reporting features leading to interactive documents.

## 1 Introduction

Bioinformatics, today, is supporting most biological and medical research projects. Bioinformatics examples will be used in the following as examples for all of data science. At the beginning, bioinformatics was mostly concerned with sequence alignment and it still is an important task. Additionally, many other tasks have developed ranging from statistical calculations to image or video analysis. In the last decades many bioinformatics tools have been developed. For example, sequence alignment can be done with a myriad of tools such as FASTA [1] and BLAST [2]. In fact, for the task of sequence alignment at least 50 tools have been developed [3]. This duplication of effort is also seen in other areas of bioinformatics. In mass spectrometry (MS), for example, there are at least ten tools for *de novo* sequencing of MS/MS spectra [4] and at least ten more for database search [5]. It is impossible to stay on top of the most recent developments for a larger amount of tool categories. Collections such as JIB Tools [6] try to organize tools into categories, but it is a manual and time consuming task for the tool editors. The tool DaTo [7] has a very comprehensive collection of tools and databases accessible via an online interface. Tools and databases for DaTo are automatically discovered but not manually annotated. Therefore, the information in DaTo is more comprehensive, but it does not provide a quality assessment of the tools and databases.

### 1.1 Quality of Computational Tools

The quality of tools in bioinformatics is often hard to assess because gold standard datasets are not available or cannot be produced for a given problem [8]. Even if good test data is available, often it is not used since formats are not agreed upon which exacerbates testing of newly developed tools. Some developments such as OpenMS [9] at least include unit tests for all their modules, but many bioinformatics tools do not include extensive testing. Instead, they may contain code smells [10], an indication that the software should be re-designed. Code smells come in many odours [11] for different types of design flaws such as Shotgun Surgery, which refers to the problem that for making a code change many parts of the project need to be changed at once. The aforementioned smell, Dead Code, and Divergent Code smells seem to be a common problem today arising from copy paste of code that work(s/ed) or appear(s/ed) to be. One reason for bioinformatics codes to be smelly is that bioinformaticians wear two heads, one for information science and one for biology or related fields. This problem has long been identified and in an attempt to overcome it, programming schools were initiated under the software carpentry umbrella [12].

### 1.2 Software Carpentry

Initiated out of frustration in 1998, software carpentry has become an organization reaching thousands of computational scientists. Its overarching aim is to teach basic lab skills for research computing. As of 2018, software and data carpentry merged their efforts (The Carpentries: https://carpentries.org/). According to their own words: “These Carpentries seek to build and grow communities of practice around computational skills development for researchers”. Today, there are about 500 accredited instructors teaching about one course on average and thereby reaching a total of around 16,000 participants. Many skills taught as part of computer science curricula are never formally disclosed to computational scientists of other disciplines who just happen to need programming skills for their daily tasks. According to Greg Wilson, a seminal figure for software carpentry, their courses increase participants’ computational skills by two-fold and make them more effective programmers in practice [13].

### 1.3 Programming Languages for Scientific Computing

**Figure 1:**
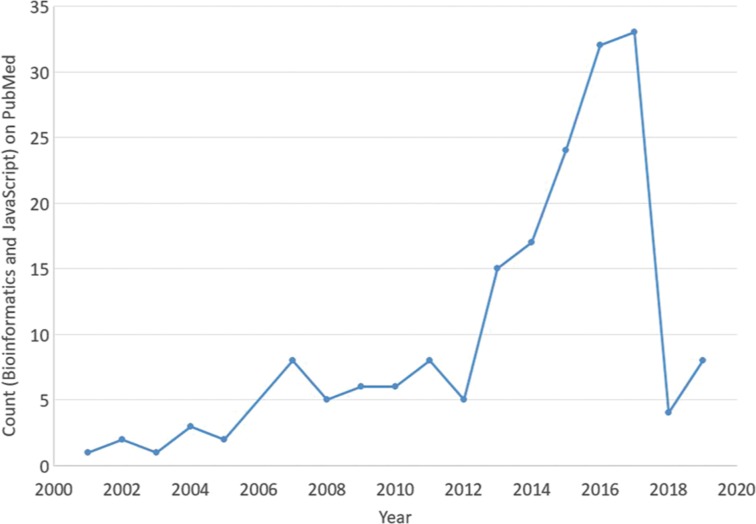
Distribution of the terms bioinformatics and JavaScript on PubMed over the last two decades.

While it is hard to assess which programming languages have been used in practice, it is likely that over the last decades any language from Ada to XOTcl has been used to solve a scientific problem computationally. In bioinformatics, Perl used to be the language of choice and it is still in use today. However, python and R are more popular than Perl at this point. This should not discard other languages and there are large scale projects developed in object oriented programming languages such as C++ (SeqAn [14], OpenMS [9]) and Java (biojava [15]). JavaScript, in the past only a client-side scripting languages in web browsers, is picking up ground and more than one hundred packages related to bioinformatics are now available on the node package manager. Also in scientific publications, JavaScript has played an increasing role in the last two decades (Figure 1).

One reason why many different programming languages are used in scientific computing is due to the background of research scientists or their advisors who are often choosing a language because of prior exposure. Popularity in the field is another factor and that should lead to a form of consolidation over time. For the field of bioinformatics that has not happened and many languages are used in parallel. In fact, many algorithms and tools have been implemented in many programming languages representing a large duplication of effort. For instance, the Smith-Waterman algorithm for local sequence alignment has been implemented in Java, C++, Perl, Python, JavaScript and other languages. Undoubtedly, the languages with the largest amount of available modules (http://www.modulecounts.com/), the biggest community (http://www.dataists.com/2010/12/ranking-the-popularity-of-programming-langauges/), and top in many other measurement categories (https://insights.stackoverflow.com/survey/2018/) is JavaScript. One recurring claim, discrediting scripting languages, involves execution speed of the resulting artifacts. This is a topic, which with just-in-time compilers available for most scripting languages such as Java and JavaScript, should be alleviated today. It should have become clear that algorithmic tweaking far surpasses benefits from choosing a particular programming language. Setting aside execution speed, for a particular problem some languages may be more suitable than others due to their inherent structure. Workflow management systems (WMS) can help piece together complex data analysis workflows using tools developed in different languages.

### 1.4 Workflow Management Systems

While using a single tool for analysis, e.g. aligning a novel sequence to known sequences, is a relatively small task (ignoring ID conversions and the large amount of available databases), the complexity of data analysis is ever increasing. On the other hand, there is a call for reproducibility of data analysis. Workflow management systems (WMS) ensure reproducibility of complex data analysis tasks. Similar to other bioinformatics tools, a large variety of WMS are available. WMS used in bioinformatics are, for example, Taverna [16], Galaxy [17], and KNIME [18] but many others exist. Some WMS include collaborative workflow development with versioning (e.g. KNIME). Most WMS allow the incorporation of new tools. For some WMS that is relatively simple (Galaxy) and for others it may be a bit more involved (KNIME). While the production of workflows can be versioned, the tools incorporated in the workflow are generally not. However, this can be achieved using Cuneiform [19], which can execute a workflow while loading specific tool revisions from git repositories. Unit testing and integration testing is not generally a part of WMS although building computational data analysis pipelines needs proper testing just like building any other software artifact. There appear to be attempts to make Galaxy WFs testable [20]. Galaxy and other WMS are running on a server and can be accessed via the internet. However, none of the WMS used in bioinformatics leverage the computational power of the server and the client or make online and offline development seamless. The internet of things has, among other developments, seen the rise of WMS to enable the analyses of sensor data.

### 1.5 Challenges

One challenge in the future of scientific computing is the transfer form workstations and desktop computers to laptops or even smaller units. Additionally, the transition away from programs to online application will change the way scientific computing is performed today. At the same time, many programs used in scientific computing are not comprehensively tested and have many alternative forms e.g. developed in different programming languages. In the following, I will suggest a system which will overcome current issues in scientific computing and streamline development of new tools and workflows. This is a call for a science community effort but also inviting the commercial sector for collaboration.

## 2 Architecture

The envisioned system will depend on a central application server, or several replicates thereof, to provide access to the IoS for development and use. For closed installations, as for example in companies, the application can be installed on a local server behind a company firewall. The application server holds the main application with several modes of accessing the system: (1) as administrator, (2) as reviewer, (3) as developer, (4) as tester, and (5) as workflow developer or user. Users and workflow developers only have access to successfully reviewed tools whereas in other access modes increasingly less strict access models apply. While access to level 5 is unrestricted, higher level access needs accreditation, for example, from providers such as ORCID. The application server does not hold data for workflows which should be linked via web-accessible resources and it does not perform computations for the user workflows. Instead, the execution of the workflows and the tools will occur elsewhere, on a trusted resource (e.g.: grid), or on all connected users which grant access to their web browser for computations, as well as public/restricted dedicated computation nodes for the IoS. In summary, the system consists of a replicated server for the main application and tools, name servers for user authentication, and different options to assign computational resources for data access and to perform computations. Thus, the IoS is a cloud based application which also facilitates access to distributed computing with the reviewed IoS tools on various cloud services. Thereby, it resembles a software as a service at least for the development of the IoS while the workflow development resembles a platform as a service structure.

## 3 Implementation

In order to overcome duplications of effort and to ensure that all tools are fully unit and integration tested, the community should pick one programming language. To allow for the development of online (server, client) and desktop applications, JavaScript needs to be the language of choice. Furthermore, JavaScript has an enormous developer base and the largest amount of existing packages. From the language perspective, it supports different programming paradigms (procedural, object oriented, and functional) such that most developers can feel at home. Choosing one programming language ensures that the community can seamlessly develop unit and integration test for the code and can build on top of comprehensively tested, dependable modules.

**Figure 2:**
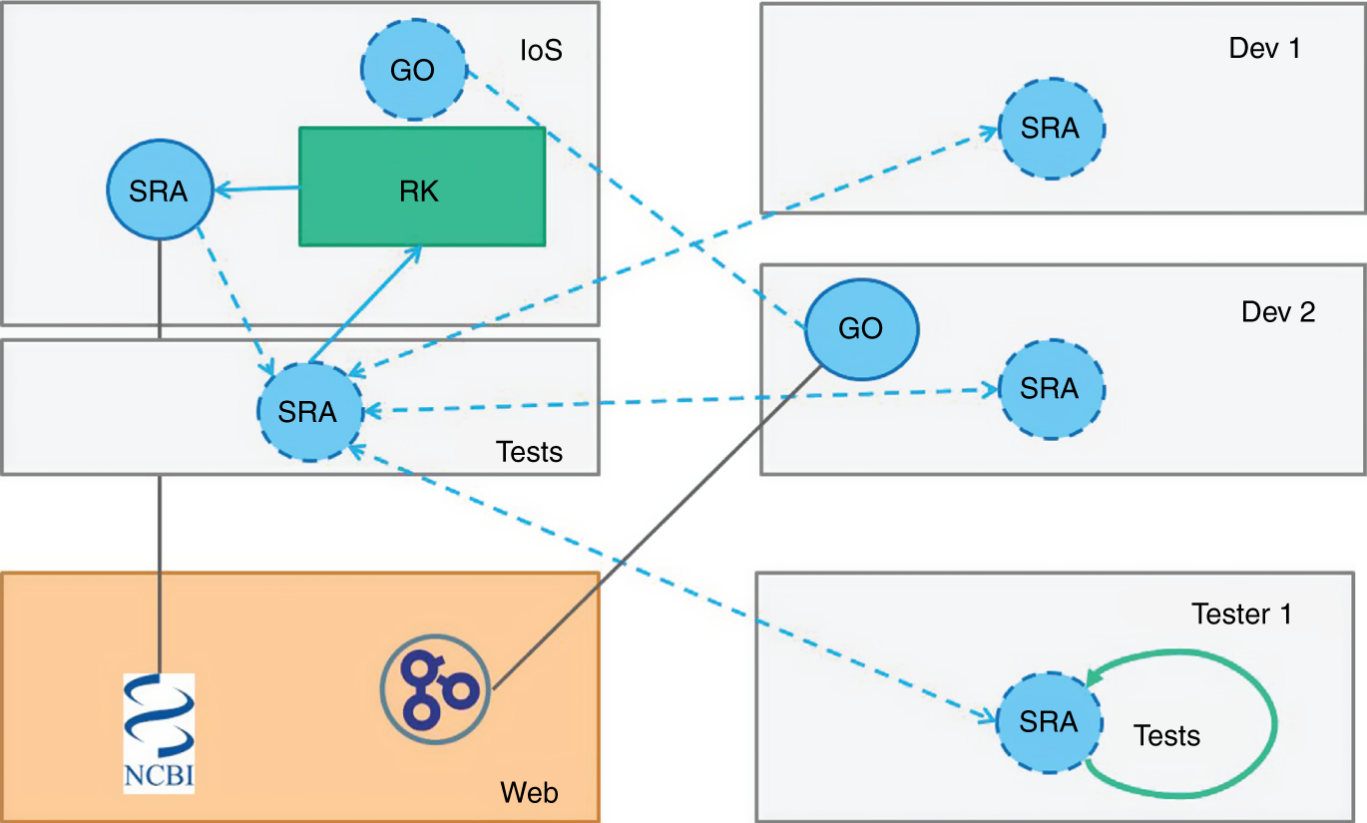
The possible process of promoting a tool to the production level. The orange box represents online services such as NCBI’s SRA and gene ontology (GO). To access such services tools can be developed (blue circles). IoS represents the production level where in this picture only SRA has a connector. The GO connector node is submitted for inclusion into the production level but has no tests yet and therefore is not scheduled for the review committee (RK; green box), yet. Developers Dev 1 and Dev 2 both pulled copies of the SRA connector node for further development while Tester 1 pulled a copy for the development of additional tests. Tests are one evidence for the decision of the review committee whether to include a tool into the production level but code review and additional steps should also be taken. The RK requests and reviews tests, as well, to ensure proper procedure. The RK is composed of researchers form the relevant field, computer scientists and other stakeholders.

It is especially important to prevent errors from propagating into critical data analysis workflows as might be used for clinical decision-making. Therefore, development and production will be separated and only workflows build solely from production level tools will be executable in the community workflow management system. Tools are promoted from the development system when they (a) pass synthetic and real world test scenarios and (b) pass the scrutiny of a self-/auto-assembled community review committee (Figure 2). Some incentives for performing reviews are to be able to in turn use the reviewed tools, extend them, and/or receive reviews for their own tools. Additionally, efforts such as developing test suites and or providing test data will be incentivised by associating them with citable digital object identifiers (DOIs).

**Figure 3:**
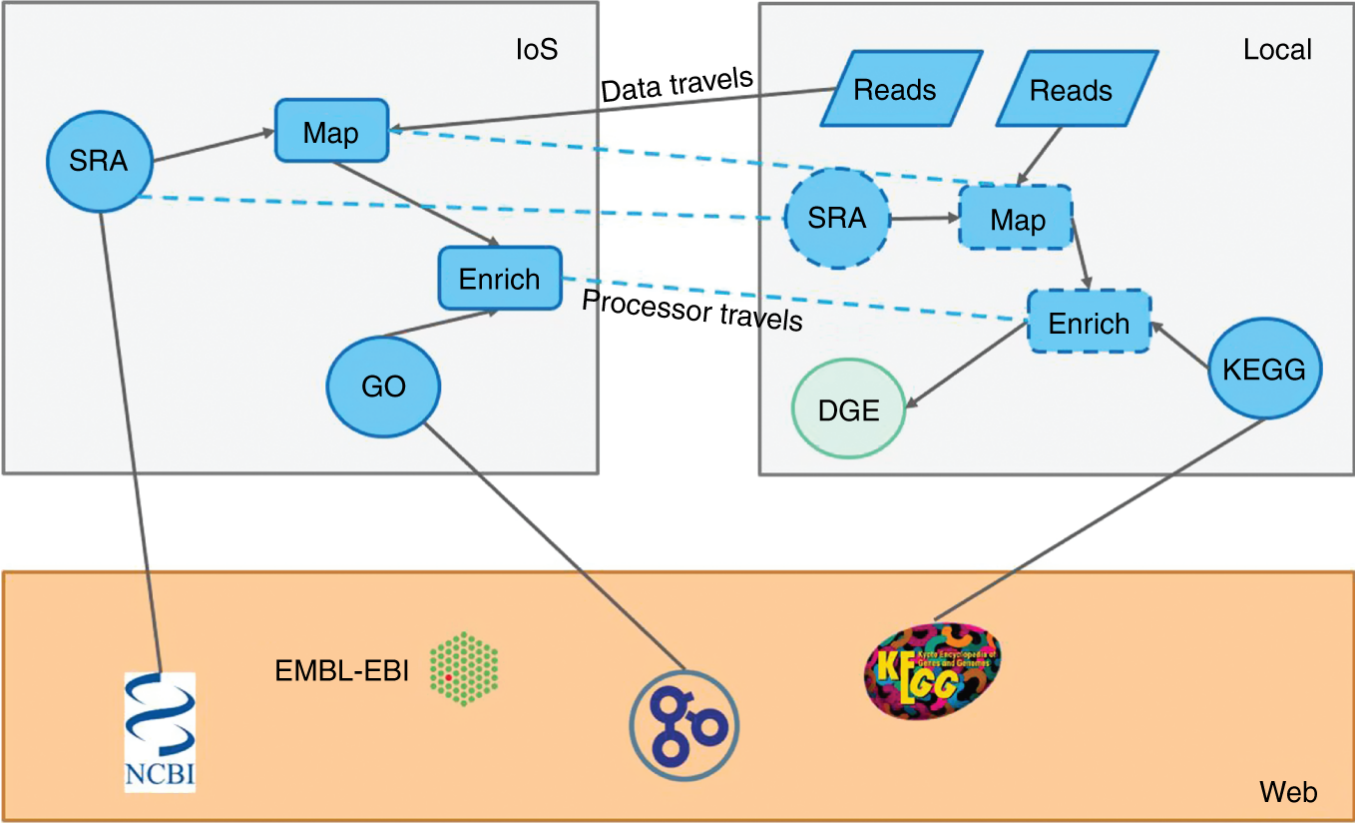
The development of data analysis workflows using a mixture of public and private data and tools. The orange box contains some public data sources (e.g.; GO [24] and KEGG [25]). Some have production level connector nodes (blue circles, IoS box) while others have private connectors (local). Processing nodes (rounded rectangles) are used to transform data. As in most WMS, Data can travel but in this system processing nodes can travel as well (dashed blue lines). Local data (blue parallelogram, e.g.; proprietary) can be processed locally so that it does not need to be uploaded. Result visualization is just another data transformation (green circle).

Successfully reviewed tools (Figure 2), are part of the production level and can then be used to build data analytics workflows (Figure 3). Self-made or not reviewed tools can be used as well, for example, by their developers but should not be acceptable for publication or for any critical data analysis. This model does not hinder new developments but will speed them up. For example, a doctoral student does not need to develop handlers or connectors for commonly used data types but can simply build a workflow using existing tools. Adding their own algorithm to the mix as an extension of existing tools or as novel tools leads to novel workflows which can then be directly used or encapsulated as modules for use in production after passing due review. Modules are also very useful for automatic algorithm selection. For example, many algorithms have been developed for exact pattern matching [21] and their performance varies with the input such that the most effective algorithm can be selected automatically. Such a scenario can be encapsulated into a module and thereby a huge reduction in complexity can be achieved shielding the users of such modules from the decision making process of which particular algorithm to use in the specific situation. This also ensures that everyone contributing to solving a particular problem with different algorithms can re-use existing tests and can directly benchmark against all other reviewed solutions using a comprehensive search space, thereby adding to the problem solution without increasing apparent complexity. Furthermore, all meaningful portions of code are citable via DOIs making the code FAIR [22] similar to already established initiatives for FAIR data [23].

Many stakeholders have an interest in data analytics and some can freely share their data while others cannot. Additionally, some developers are working for profit while others do not. The envisioned platform accommodates all these and other constraints. For example, data can stay local and can be processed on site by downloading processors to the local version of the IoS. This is seamless with non-for-profit processing nodes but for commercial nodes, different licensing models (e.g. prepaid, use once and burn) need to ensure proper handling. For commercial purposes local modules not submitted to the IoS may present a business case, but passing the tool through the review process and making it commercial may be more advantageous since the community may not trust unreviewed tools. This setup and the use of JavaScript hold other promises such as the sharing of computational resources via processing in connected web browsers, as exemplified by QMachine [26]. Automatic encapsulation of tools or complete workflows into executables facilitates automatic parallel execution on larger computer infrastructures.

Reporting is an important part of a computational data analytics workflow. In fact, since the workflow already defines all inputs, data transformations, and outputs, reporting is fully traceable from any report item to the underlying raw data. The aim is to integrate workflow outputs into documents which can be collaboratively developed online. The outputs will allow direct access to the reasoning down to the raw data from within the document. This type of interactive document completely encapsulates the intent and the approach taken and, together with the embedded IoS workflows, ensures reproducibility.

For future development of the internet of science, a public repository has been initialized: https://bitbucket.org/allmer/ios/src/master/. Developers of the IoS system need to be approved to gain access but read access is public. The IoS will be presented several times in 2019 and towards the beginning of 2020, a conference is planned to bring together interested parties and to grow the community.

## 4 Discussion

The main aim of the internet of science is to re-establish collaboration as the first principle of science. The IoS enables collaborative work on developing and testing software, developing and testing data analysis workflows, and joint reporting. It binds together all stakeholders while enabling the tracking of individual and joint efforts via DOIs. The IoS embraces FAIR code and appreciates FAIR data. Documents developed using IoS provide access to data, the IoS workflow for data analysis and in turn all code involved as well as to the document thus making information also FAIR.
